# Antidiabetic Potential of Polysaccharides from Indian Tea Cultivars Using *in vitro* and *in vivo* Evaluation

**DOI:** 10.17113/ftb.64.03.26.9794

**Published:** 2026-09-15

**Authors:** Nishanth Pitcham, Nivedidha Arumugam, Hariram Natarajan

**Affiliations:** Department of Biotechnology, Kalasalingam Academy of Research and Education, Krishnankoil, 626126 Tamil Nadu, India

**Keywords:** tea polysaccharides, Indian tea cultivars, antidiabetic activity, enzyme inhibition, functional food, glycaemic control

## Abstract

**Research background:**

Natural polysaccharides have gained increasing attention because of their potential role in the management of diabetes mellitus. Tea is a widely consumed beverage containing structurally diverse polysaccharides that contribute to its health benefits. However, polysaccharides from Indian tea cultivars remain insufficiently explored, particularly regarding their structural characteristics and antidiabetic potential.

**Experimental approach:**

Fresh leaves from six Indian tea cultivars (TV-17, TV-22, Teen Ali, TRF-1, UPASI-9 and UPASI-28) were screened for polysaccharide yield using hot-water extraction followed by ethanol precipitation. Based on their higher polysaccharide content, Teen Ali and UPASI-28 were selected for further structural characterisation by Fourier-transform infrared spectroscopy and scanning electron microscopy. Their antidiabetic potential was evaluated through α-amylase and α-glucosidase inhibition assays and further validated in an alloxan-induced diabetic mouse model.

**Results and conclusions:**

The isolated polysaccharides showed characteristic structural features of carbohydrate-rich biomolecules and had distinct morphological properties. Both samples demonstrated significant inhibitory activity against major carbohydrate-hydrolysing enzymes, indicating their potential to regulate glucose metabolism. *In vivo* studies further confirmed their antidiabetic efficacy through improved glycaemic control, recovery from diabetes-associated body mass loss, and recovery of tissue architecture in treated groups. The observed biological activity may be associated with structural attributes such as hydroxyl groups and uronic acid content, which could enhance interactions with target enzymes.

**Novelty and scientific contribution:**

This study provides a comparative investigation of polysaccharides derived from Indian tea cultivars by integrating structural characterisation with both *in vitro* and *in vivo* antidiabetic evaluations. The findings contribute to the understanding of tea polysaccharides as potential natural therapeutic agents for diabetes management and highlight the biomedical relevance of underexplored Indian tea varieties.

## INTRODUCTION

Diabetes mellitus remains one of the most pressing global health challenges due to its rising prevalence and its association with complications such as cardiovascular disease, nephropathy and retinopathy (*1*). Although several pharmacological therapies are available, many are limited by side effects, high cost or reduced long-term efficacy (*2*), prompting an increasing interest in natural, safer alternatives for glycaemic management. Plant-derived polysaccharides have emerged as multifunctional bioactive compounds with immunomodulatory, antioxidant and antidiabetic properties (*3*). Owing to their diverse structural features, such as uronic acids, protein-bound fractions and varied branching patterns, these macromolecules can modulate glucose metabolism through multiple mechanisms, including inhibition of carbohydrate-digesting enzymes, enhancement of insulin sensitivity, protection of pancreatic β-cells and regulation of gut microbiota (*4*). Their low toxicity and widespread occurrence make them promising candidates for functional foods and nutraceutical development.

Tea (*Camellia sinensis* (L.) Kuntze) is consumed worldwide, and although its health benefits are commonly attributed to polyphenols, recent studies highlight tea polysaccharides as key contributors to its therapeutic potential. Tea polysaccharides show considerable structural variation depending on tea type, cultivar and processing method, leading to differences in monosaccharide composition, molecular mass and acid substitution that correlate with biological activity (*5*). For example, tea polysaccharides from pu-erh tea have shown hypoglycaemic effects in alloxan-induced diabetic mice by enhancing antioxidant enzymes superoxide dismutase (SOD) glutathione peroxidase (GSH-Px) and reducing lipid peroxidation malondialdehyde (MDA) (*6*). Similarly, tea polysaccharides have been shown to exert hypoglycaemic and hypolipidaemic effects in type 2 diabetic rat models through modulation of gut microbiota and metabolic pathways (*7*). Dietary bioactive compounds such as polysaccharides have been shown to improve glycaemic control in diabetic conditions (*8*). Nevertheless, few studies have examined the structural characteristics and biochemical properties of tea polysaccharides isolated from Indian varieties of tea (*9*). Extraction conditions also affect the efficacy of tea polyphenols, with green tea leaf and flower polysaccharides differing in monosaccharide composition and *in vitro* α-glucosidase/α-amylase inhibition depending on the extraction method (*3*).

Despite these advances, tea polyphenols from Indian tea cultivars, particularly Assam and South Indian varieties, remain underexplored. These regions possess unique genetic backgrounds and agro-climatic conditions that influence tea leaf chemistry, yet few comparative studies have investigated tea polyphenols from these cultivars. Previous studies have demonstrated that selenium-enriched tea polysaccharides can improve insulin resistance and reduce oxidative stress in experimental models (*10*). However, the contribution of selenium and polysaccharide fractions to these biological effects remains difficult to distinguish, highlighting the need to investigate non-supplemented tea polysaccharides from different cultivars. Moreover, most existing reports lack an integrated approach that combines *in vitro* enzyme inhibition, *in vivo* antidiabetic evaluation, and histopathological validation.

To address these gaps, the present study investigates polysaccharides from two Indian tea cultivars, Teen Ali (Assam) and UPASI-28 (South India) to evaluate their antidiabetic potential using α-amylase and α-glucosidase inhibition assays, alloxan-induced diabetic mouse models, and histological analyses to establish their efficacy and therapeutic relevance. Understanding the functional capabilities of regional tea polysaccharides is important for using a natural approach to control blood glucose levels. Accordingly, the current research evaluates the ability of tea polysaccharides isolated from Indian varieties through *in vitro* enzyme inhibition studies and *in vivo* animal experiments. Enzyme inhibition studies were conducted alongside *in vivo* experiments to provide a comprehensive assessment of both the biochemical properties and physiological significance of the obtained polysaccharides. In the present study, six Indian tea cultivars were initially screened for polysaccharide yield and preliminary antidiabetic potential. Based on the screening results, the two cultivars with the highest polysaccharide content (Teen Ali and UPASI-28) were selected for detailed physicochemical characterisation and biological evaluation. Thus, the study was designed as a two-stage investigation, consisting of cultivar screening followed by a comprehensive assessment of the most promising polysaccharide-rich cultivars.

## MATERIALS AND METHODS

### Tea samples

Fresh tea leaves from six cultivars were collected from experimental tea plantations in Assam, India (TV-17, TV-22 and Teen Ali) and Tamil Nadu, India (TRF-1, UPASI-9 and UPASI-28). Samples were collected during the active growing season and transported to the laboratory under ambient conditions for further processing. The leaves were washed thoroughly with distilled water, shade-dried at room temperature (25–28 °C) and ground to a fine powder using a sterile mortar and pestle. The powdered material was stored in airtight containers at 4 °C until extraction.

### Polysaccharide extraction

Polysaccharides were extracted following a modified hot-water extraction and ethanol precipitation protocol. Dried powdered tea leaves (10 g) were suspended in 200 mL of distilled water at 95 °C for 2 h. The extract was filtered and concentrated, and polysaccharides were precipitated by adding 96 % ethanol (analytical grade; Merck Life Science Pvt. Ltd., Mumbai, India) to a final volume fraction of 86 %. The mixture was centrifuged at 7500×*g* for 10 min at 4 °C (5430R; Eppendorf SE, Hamburg, Germany). The precipitate was washed twice with 80 % ethanol to remove mono- and oligosaccharides, then dried and dissolved in distilled water. The mixture was kept at 4 °C overnight to ensure complete precipitation and then centrifuged at 7500×*g* for 10 min. The recovered precipitate was washed twice with 80 % ethanol, dried under vacuum, and dissolved in distilled water for further analyses. No additional purification procedures were applied. The extraction protocol was adapted from Hu *et al.* (*5*) with minor modifications.

### Polysaccharide quantification (phenol–sulfuric acid method)

Polysaccharide mass fraction was quantified using the phenol (analytical grade; Merck Life Science Pvt. Ltd.)–sulfuric acid (95–98 %; Merck Life Science Pvt. Ltd.) colorimetric assay with soluble starch (HiMedia Laboratories Pvt. Ltd., Mumbai, India) as the calibration standard, following Stewart (*11*) with modifications. A starch standard curve was prepared using 0, 20, 40, 60, 80 and 100 µg/mL starch solutions. To each well of a 96-well plate, 50 µL of either sample or standard were added, followed by 30 µL of 5 % phenol and 150 µL of concentrated sulfuric acid. Plates were incubated at 90 °C for 5 min and then cooled to room temperature. Absorbance was measured at 492 nm using a Multiskan GO microplate reader (Thermo Fisher Scientific, Vantaa, Finland).

A linear regression equation was obtained using the following equation:


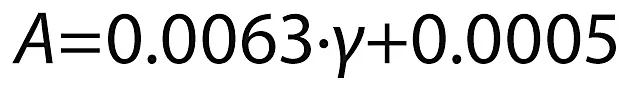
 /1/

where *A* is the absorbance and *γ* is starch concentration (µg/mL). Sample absorbances were converted into starch-equivalent concentrations, and final polysaccharide yield was expressed as mg of starch equivalents per gram of dry leaf mass (mg/g) using the following equation:


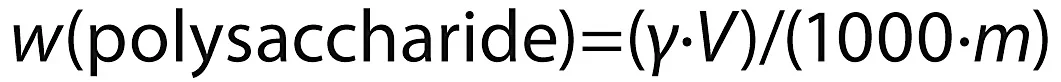
 /2/

where *γ* is sample concentration (μg/mL), *V* is the final volume of the sample (mL), and *m* is the dry mass of the sample (g).

Where applicable, samples were diluted to fit within the linear range of the standard curve. All measurements were performed in triplicate.

### FTIR analysis

The structural characteristics of the extracted polysaccharides were analysed using Fourier-transform infrared spectroscopy (FTIR; IRTracer-100; Shimadzu, Kyoto, Japan). Dried polysaccharide samples were mixed with spectroscopic-grade potassium bromide (Merck Life Science Pvt. Ltd.), compressed into pellets, and scanned over the wavelength range of 4000–400 cm^−1^ at a resolution of 4 cm^−1^. The obtained spectra were used to identify characteristic functional groups associated with tea polysaccharides.

### Scanning electron microscopy

The surface morphology of tea polysaccharides was examined using scanning electron microscopy. Dried polysaccharide samples were mounted on aluminium stubs with double-sided conductive carbon tape and sputter-coated with a thin layer of gold to improve conductivity. The coated samples were observed using a scanning electron microscope (JSM-IT200; JEOL Ltd., Tokyo, Japan) operated at an accelerating voltage of 10 kV. Micrographs were recorded at magnifications of 250, 500, 1000 and 2500× to evaluate particle morphology, surface characteristics, aggregation patterns and porosity of the polysaccharide samples.

### In vitro antidiabetic activity

#### α-Amylase inhibition assay

A 1 % (*m*/*V*) starch solution was prepared by dissolving 1 g of soluble starch in 100 mL of 20 mM phosphate buffer (pH=6.9) containing 6.7 mM NaCl. The α-amylase (HiMedia Laboratories Pvt. Ltd.) solution (1 U/mL) was prepared in the same buffer. Polysaccharides were dissolved in a minimal volume of dimethyl sulfoxide (DMSO; HiMedia Laboratories Pvt. Ltd.) to a final *φ*(DMSO)<1 %, and diluted with phosphate buffer to obtain final standard solution concentrations of 10, 20, 40, 60, 80 and 100 µg/mL. In a test tube, 200 µL of enzyme solution were mixed with 200 µL of polysaccharide solution and preincubated at 37 °C for 10 min. Then, 200 µL of starch solution were added to initiate the reaction and the mixture was further incubated at 37 °C for 10 min. The reaction was terminated by adding 200 µL of 3,5-dinitrosalicylic acid (DNS; SRL Chemicals, Mumbai, India) reagent, prepared by dissolving 1 g of 3,5-dinitrosalicylic acid and 30 g of sodium potassium tartrate (SRL Chemicals) in 20 mL of 2 M sodium hydroxide (Merck Life Science Pvt. Ltd.) and making up the volume to 100 mL. The tubes were then placed in a boiling water bath for 5 min, cooled to room temperature and diluted with 2 mL of distilled water. Absorbance was measured at 540 nm using a UV-visible spectrophotometer (UV-1800; Shimadzu). Acarbose (10–100 µg/mL; Sigma-Aldrich, Merck, St. Louis, MO, USA) served as the positive control. The blank was prepared by replacing the enzyme solution with 200 µL of distilled water, while the control contained enzyme and substrate without inhibitor. All assays were performed in triplicate (*N*=3) and the inhibition percentage was calculated using the following equation:


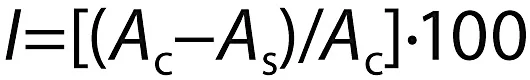
 /3/

where *I* is the inhibition percentage, *A*_c_ is the absorbance of the control and *A*_s_ is the absorbance of the sample. Values were calculated as mean±average standard error of the mean of three repetitions (*12*, *13*).

#### α-Glucosidase inhibition assay

Inhibition of α-glucosidase (Sigma-Aldrich, Merck) activity was determined using yeast α-glucosidase and *p*-nitrophenyl-α-d-glucopyranoside (Sigma-Aldrich, Merck) as described by Kim *et al.* (*14*). Acarbose and polysaccharides from tea plants (10, 20, 40, 60, 80 and 100 µg/mL) were added to 50 μL of α-glucosidase (1 U/mL) prepared in 0.1 M phosphate buffer (pH=6.9) and then 250 μL of 0.1 M phosphate buffer were added to obtain a final concentration of 0.5 to 5.0 mg/mL. The mixture was preincubated at 37 °C for 20 min. After preincubation, 10 μL of 10 mM *p*-nitrophenyl-α-d-glucopyranoside prepared in 0.1 M phosphate buffer (pH=6.9) was added and then incubated at 37 °C for 30 min. The reactions were stopped by adding 650 μL of 1 M sodium carbonate, and the absorbance was measured using a spectrophotometer (UV-1800; Shimadzu) at 405 nm. An α-glucosidase inhibitor (acarbose) was used as a standard. The percentage of inhibition of α-glucosidase was determined by Eq. 3. Values are expressed as mean±standard error of the mean (*N*=3) (*12*, *13*). The concentration of each polysaccharide sample required to inhibit 50 % of enzyme activity (IC_50_) was calculated by plotting percentage inhibition against the logarithm of inhibitor concentration and fitting the data to a sigmoidal dose–response curve using nonlinear regression (GraphPad Prism v. 9.0) (*15*). All experiments were performed in triplicate, and results are presented as mean value±standard error of the mean.

### In vivo studies

Healthy male BALB/c mice (average body mass (28±2) g) were obtained from the institutional animal facility. Only male mice were used in this study to minimise biological variability. Sex-based differences were not evaluated and represent a limitation of the study. The animals were housed under standard laboratory conditions ((22±2) °C, 12 h light/12 h dark cycle, relative humidity 50–60 %) with free access to standard pellet diet and water *ad libitum*. All experimental protocols were approved by the Institutional Animal Ethics Committee Arulmigu Kalasalingam College of Pharmacy, Krishnankoil (IAEC), India, and conducted in accordance with CPCSEA/NIH guidelines for the care and use of laboratory animals (*16*).

Diabetes was induced by a single intraperitoneal injection of alloxan monohydrate (Sigma-Aldrich, Merck) (150 mg per kg body mass) freshly prepared in sterile normal saline. Mice were fasted overnight (12 h) before injection, with free access to water. After 72 h, blood glucose concentrations were measured using tail vein blood and a glucometer. Mice with fasting blood glucose ≥200 mg/dL were considered diabetic and included in the study. Fasting blood glucose concentrations were measured on days: 0 (baseline, post-induction), 7, 14 and 21 using a handheld glucometer, with blood collected from the tail vein after overnight fasting. Body mass was recorded on the same days to monitor changes during treatment.

At the end of the 21-day treatment period, animals were fasted overnight and euthanised under IAEC-approved ethical procedures using an overdose of anaesthesia. The pancreas, liver and kidney were immediately excised, washed with ice-cold normal saline, fixed in 10 % neutral buffered formalin for 24–48 h, processed by routine paraffin embedding, sectioned at approx. 5 μm thickness, stained with haematoxylin and eosin (H&E), and examined under a light microscope (CX23; Olympus Corporation, Tokyo, Japan) for histopathological alterations.

### Experimental design

Mice were randomly divided into nine groups (*N*=6 per group) as follows: (*i*) group I (control): normal mice receiving vehicle only, (*ii*) group II (diabetic control): alloxan-induced diabetic mice receiving vehicle only, (*iii*) group III (standard): diabetic mice treated with metformin (150 mg/kg body mass/day, orally), (*iv*) group IV (low dose): diabetic mice treated with polysaccharides from Teen Ali (10 mg/kg body mass/day, orally), (*v*) group V (medium dose): diabetic mice treated with polysaccharides from Teen Ali (50 mg/kg body mass/day, orally), (*vi*) group VI (high dose): diabetic mice treated with polysaccharides from Teen Ali (100 mg/kg body mass/day, orally), (*vii*) group VII (low dose): diabetic mice treated with polysaccharides from UPASI-28 (10 mg/kg body mass/day, orally), (*viii*) group VIII (medium dose): diabetic mice treated with polysaccharides from UPASI-28 (50 mg/kg body mass/day, orally), and (*ix*) group IX (high dose): diabetic mice treated with polysaccharides from UPASI-28 (100 mg/kg body mass/day, orally). Six Indian tea cultivars were initially screened for polysaccharide yield using the phenol-sulfuric acid method and for their *in vitro* antidiabetic potential through α-amylase and α-glucosidase inhibition assays. Among the six cultivars, Teen Ali and UPASI-28 exhibited the highest polysaccharide yields, expressed on dry mass basis as SE (55 and 45 mg/g, respectively) together with the strongest inhibitory activities against both enzymes. Based on these preliminary screening results, these two cultivars were selected for detailed physicochemical characterisation and comprehensive *in vivo* antidiabetic evaluation, while the remaining cultivars served as the initial screening group.

Dose selection was based on previous investigations demonstrating the safety and biological efficacy of food-derived polysaccharides within this dosage range in experimental diabetic models (*5*, *10*, *16*). All treatments were administered orally once daily for 21 consecutive days. Doses were calculated individually based on an average body mass of 28 g.

### Statistical analysis

Data are presented as mean value±standard error of the mean. Before statistical analysis, data normality was assessed using the Shapiro-Wilk test. As all datasets followed a normal distribution (p>0.05), statistical comparisons among groups were performed using one-way analysis of variance (ANOVA) followed by Tukey’s multiple comparison test. Differences were considered statistically significant at p<0.05. Statistical analyses were conducted using GraphPad Prism v. 9.0 (*15*).

## RESULTS AND DISCUSSION

### Extraction and quantification

Quantification of total polysaccharides from all tea cultivars revealed differences in carbohydrate content. Fig. 1a shows the standard curve generated using the phenol-sulfuric acid assay, with starch standards ranging from 0 to 100 µg/mL. The obtained linear regression equation was:


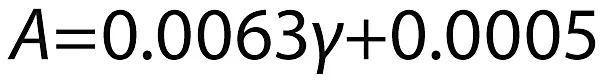
 /4/

**Fig. 1:**
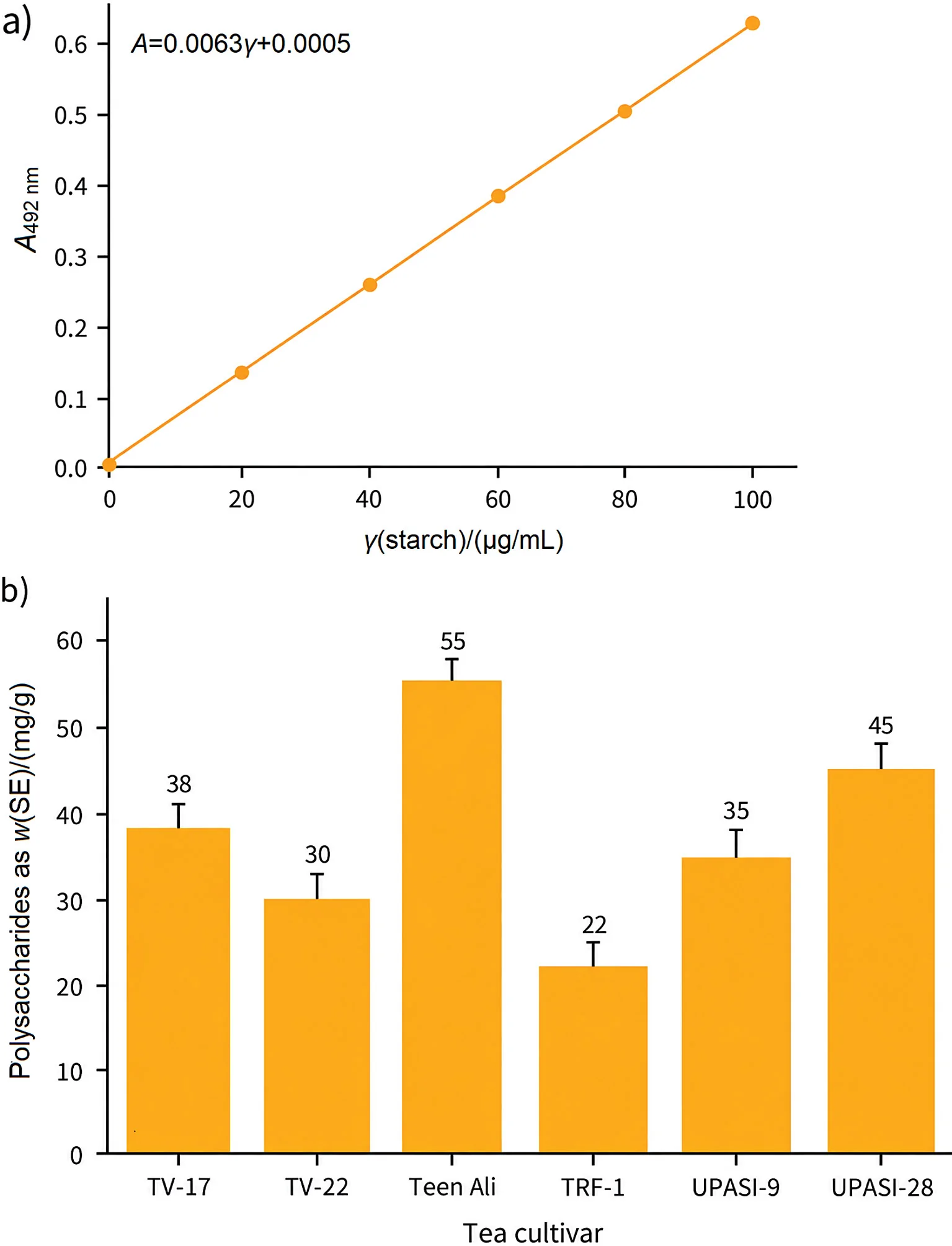
Quantification of polysaccharides: a) standard curve of starch obtained using the phenol-sulfuric acid method, and b) polysaccharide yield of six tea cultivars expressed as mg starch equivalents (SE) per g dry mass

where *A* is the absorbance measured at 492 nm and *γ* is the starch concentration (µg/mL).

Fig. 1b shows the mass fractions of extracted polysaccharides from six different tea cultivars, expressed in mg starch equivalents (SE) per g dry mass. For tea cultivars TV-17, TV-22, Teen Ali, TRF-1, UPASI-9 and UPASI-28, the obtained mass fractions of polysaccharides, on dry mass basis, were 38, 30, 55, 22, 35 and 45 mg/g, respectively. These results indicate that Teen Ali and UPASI-28 have higher mass fractions of polysaccharides than the other cultivars, which further supports their use in exploring antidiabetic potential.

The variability in polysaccharide production among the different varieties suggests the influence of genotype and environment on carbohydrate content, as previously reported in studies on the effects of environment and variety on the composition of polysaccharides in tea plants (*7*, *16*).

The variability in polysaccharide yield observed among the tea cultivars is consistent with previous reports indicating that tea genotype, geographical origin, environmental conditions and processing methods significantly influence polysaccharide accumulation. Guo *et al.* (*17*) reported considerable variation in extraction yields among twelve Chinese tea varieties, with polysaccharide yields ranging from 1.81 to 6.38 % depending on tea category and compositional characteristics. Similarly, Hu *et al.* (*5*) emphasised that differences in cultivar background and extraction conditions strongly affect the structural composition and biological functionality of tea polysaccharides. The comparatively higher polysaccharide content observed in Teen Ali and UPASI-28 may therefore reflect cultivar-specific metabolic differences associated with carbohydrate biosynthesis and storage.

### FTIR spectra of tea polysaccharides

FTIR analysis of the various tea cultivars revealed similar polysaccharide characteristics across all samples. For instance, the broader band for the O-H stretch was observed around 3200-3400 cm^−1^, while C-H stretching for the samples was around 2920-2940 cm^−1^. Teen Ali and UPASI-28 also displayed much weaker amide bands around 1630-1650 cm^−1^, indicating much lower levels of protein contamination and higher polysaccharide purity (Fig. 2). In addition, the band around 1730-1750 cm^−1^ corresponding to uronic acids was stronger in the Teen Ali and UPASI-28 cultivars, while the fingerprint region around 1000-1200 cm^−1^ for glycosidic linkages was very distinct in all samples. The structural integrity of the samples was particularly evident for Teen Ali and UPASI-28, which may contribute to the enhanced bioactivity observed in these samples, associated with stronger antidiabetic effects of the polysaccharides.

**Fig. 2:**
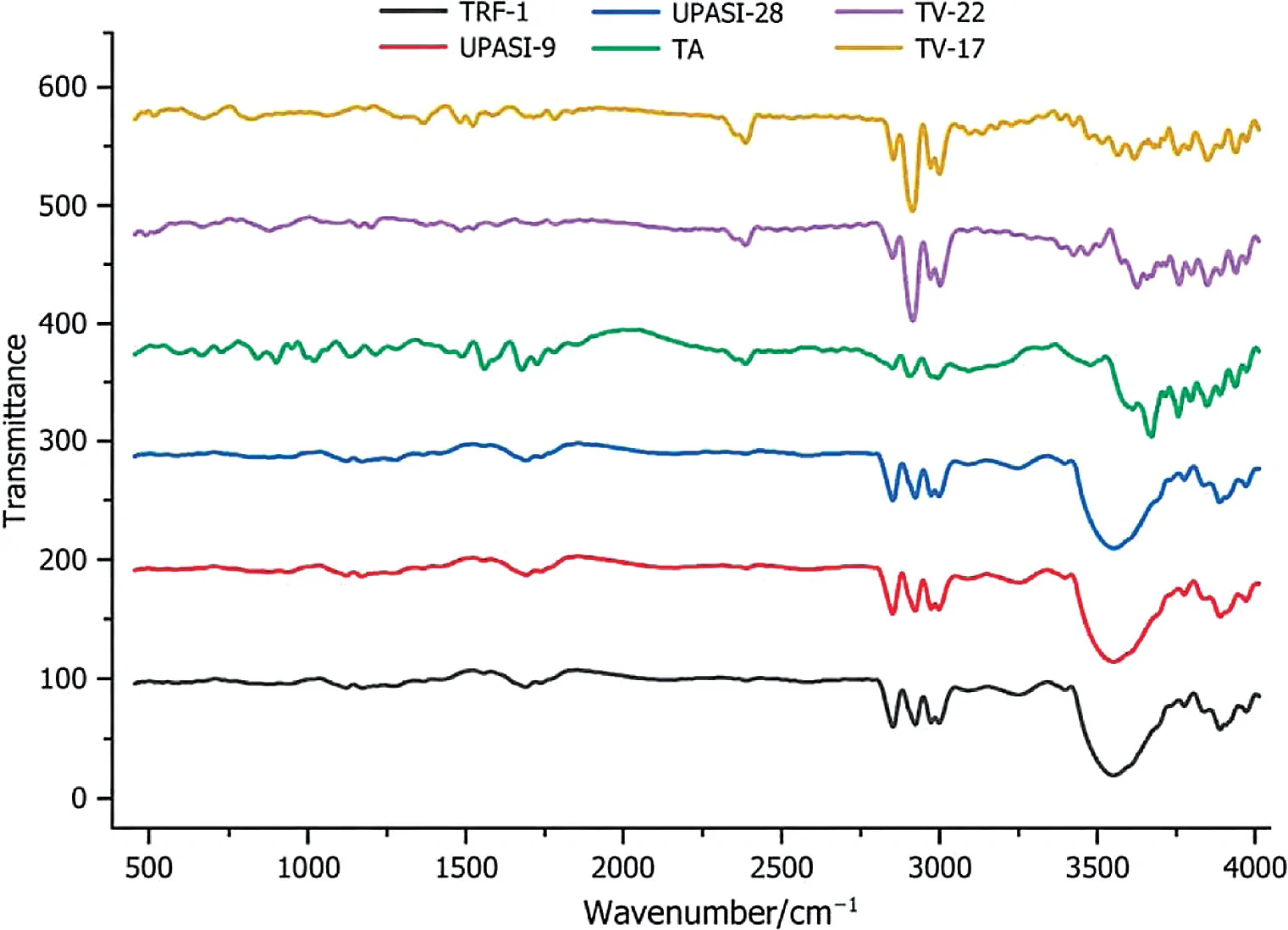
FTIR spectra of polysaccharides extracted from different tea cultivars showing characteristic functional groups, including hydroxyl, C–H, amide, uronic acid, and glycosidic linkages

Moreover, the presence of uronic acids increases the strength of electrostatic interactions and enzyme-binding efficiency, thus making them effective inhibitors. According to recent findings, polysaccharides containing uronic acids and hydroxyl groups are more efficient enzyme inhibitors than others, owing to better molecular interactions and greater conformational flexibility (*16*, *17*). These structural features collectively support the enhanced enzyme inhibitory activity observed in tea polysaccharide samples. Similar structural characteristics have been reported for tea polysaccharides isolated from Chinese green tea and pu-erh tea varieties, where strong hydroxyl and glycosidic linkage signals were associated with enhanced biological activities. Guo *et al.* (*17*) reported that tea polysaccharides containing abundant uronic acid residues had improved antioxidant and enzyme inhibitory properties. The stronger uronic acid-associated absorption bands observed in Teen Ali and UPASI-28 may therefore contribute to their superior antidiabetic activity through enhanced interactions with digestive enzymes.

### Morphological characterisation of tea polysaccharides

Scanning electron microscopy showed morphological differences between polysaccharides isolated from various tea cultivars (Fig. S1). The images showed irregular, agglomerated and porous structures with significant differences in surface topography and structural arrangement. Polysaccharides from TRF-1 and UPASI-9 had tight, irregular flakes with a high degree of aggregation, implying strong intramolecular bonding and limited surface porosity. In contrast, TV-17 and TV-22 had amorphous, heterogeneous structures with smooth surfaces and poorly organised morphology, suggesting weaker intermolecular cohesion. Remarkably, UPASI-28 had a highly porous, honeycomb-like structure, providing a larger surface area and promoting enzyme interactions. Likewise, polysaccharides from Teen Ali possessed granular, clustered structures with moderate surface roughness, implying higher reactivity and better binding affinity towards enzymes.

Variation in the structure of tea polysaccharides can greatly affect their biological properties, especially enzyme inhibition. Porous and loosely structured polysaccharides, such as those from UPASI-28 and Teen Ali, can interact more effectively with α-amylase and α-glucosidase because of their larger surface area and greater substrate accessibility. It has also been reported that porous, unevenly structured polysaccharides provide better biological activity owing to increased interaction with target enzymes and increased molecular diffusion. The higher antidiabetic activity of polysaccharides from Teen Ali and UPASI-28 can thus be attributed to the improved microstructure of these polysaccharides. Similar structure–bioactivity relationships have been reported for plant polysaccharides, where porous morphology enhances enzyme accessibility and functional performance (*16*). Morphological properties are increasingly recognised as important determinants of polysaccharide bioactivity. Previous investigations have shown that porous and irregular polysaccharide structures provide greater surface area for molecular interactions and facilitate improved accessibility to target enzymes. Zhu *et al.* (*18*) demonstrated that tea polysaccharide fractions possessing more porous architectures exhibited stronger hypoglycaemic activity than compact structures. The highly porous honeycomb-like morphology observed in UPASI-28 and the granular surface organisation of Teen Ali may therefore contribute to their enhanced interaction with α-amylase and α-glucosidase, resulting in improved inhibitory activity.

### In vitro antidiabetic activity of tea polysaccharides

The studies on the inhibition of α-amylase clearly demonstrated a concentration-dependent response for the inhibitory activity of all tea samples. Among these, Teen Ali and UPASI-28 showed potent inhibitory activity of 90.4 and 89.1 %, respectively, at 100 µg/mL, compared to 86.8 % for the standard drug acarbose (Table 1). The IC_50_ values calculated using non-linear regression for Teen Ali and UPASI-28 were 32.8 and 33.5 µg/mL, respectively, which were significantly lower than the IC_50_ of acarbose (42.3 µg/mL) and much lower than the IC_50_ values for the other varieties (55-58 µg/mL). The one-way ANOVA showed significant differences among all treatment groups (p<0.0001). Statistical differences among treatments were determined using Tukey's *post hoc* test, in which different letters in superscript indicate statistically significant differences (p<0.05). The polysaccharides from Teen Ali and UPASI-28 exhibited significantly greater inhibitory capacity than those from other varieties and acarbose, a common medication used to treat diabetes. These results confirm the strong inhibitory potential of tea polysaccharides against carbohydrate-digesting enzymes.

**Table 1:** *In vitro* inhibition of α-amylase and α-glucosidase by different concentrations of tea polysaccharides

	Tea cultivar						
*γ*(tea polysaccharide)/(µg/mL)	TV-17	TV-22	Teen Ali	TRF-1	UPASI-9	UPASI-28	Acarbose
*In vitro* α-amylase inhibitory activity/%							
10	(8.9±0.4)ᶜ	(9.2±0.5)ᶜ	(12.5±0.6)ᵃ	(9.6±0.5)ᶜ	(9.8±0.5)ᶜ	(11.7±0.6)ᵇ	(12.3±1.1)ᵃ
20	(20.3±0.7)ᶜ	(21.5±0.8)ᶜ	(32.8±0.9)ᵃ	(22.1±0.8)ᶜ	(23.4±0.9)ᶜ	(31.4±0.8)ᵇ	(25.4±1.1)ᵇ
40	(35.6±0.8)ᶜ	(36.9±0.9)ᶜ	(56.7±1.0)ᵃ	(38.2±0.9)ᶜ	(39.5±0.9)ᶜ	(55.2±0.9)ᵇ	(39.1±1.2)ᶜ
60	(48.7±1.0)ᶜ	(49.8±1.0)ᶜ	(73.5±1.2)ᵃ	(51.2±1.0)ᶜ	(52.6±1.0)ᶜ	(72.1±1.1)ᵇ	(52.6±1.4)ᶜ
80	(59.2±1.1)ᶜ	(60.5±1.1)ᶜ	(85.6±1.1)ᵃ	(61.9±1.1)ᶜ	(63.1±1.1)ᶜ	(84.7±1.0)ᵇ	(70.2±1.5)ᵇ
100	(67.8±1.2)ᶜ	(68.9±1.2)ᶜ	(90.4±1.2)ᵃ	(69.5±1.2)ᶜ	(70.7±1.2)ᶜ	(89.1±1.2)ᵃ	(86.8±0.8)ᵇ
*In vitro* α-glucosidase inhibitory activity/%							
10	(10.2±0.4)ᶜ	(10.8±0.5)ᶜ	(14.3±0.6)ᵃ	(11.5±0.5)ᶜ	(12.1±0.5)ᶜ	(13.5±0.5)ᵇ	(15.2±1.0)ᵃ
20	(23.6±0.7)ᶜ	(24.2±0.8)ᶜ	(35.7±0.9)ᵃ	(25.1±0.8)ᶜ	(26.4±0.8)ᶜ	(34.2±0.8)ᵇ	(28.7±1.2)ᵇ
40	(38.4±0.9)ᶜ	(39.6±0.9)ᶜ	(60.8±1.0)ᵃ	(40.8±0.9)ᶜ	(42.2±0.9)ᶜ	(59.4±0.9)ᵇ	(42.4±1.3)ᶜ
60	(51.6±1.0)ᶜ	(52.7±1.0)c	(76.4±1.1)ᵃ	(53.9±1.0)ᶜ	(55.4±1.0)ᶜ	(75.1±1.0)ᵇ	(55.9±1.4)ᶜ
80	(62.5±1.1)ᶜ	(63.8±1.1)ᶜ	(87.5±1.0)ᵃ	(65.1±1.1)ᶜ	(66.8±1.1)ᶜ	(86.2±1.0)ᵇ	(73.6±1.5)ᵇ
100	(70.8±1.2)ᶜ	(71.9±1.2)ᶜ	(92.7±1.2)ᵃ	(72.4±1.2)ᶜ	(73.6±1.2)ᶜ	(91.4±1.1)ᵃ	(89.1±0.9)ᵇ

Values are expressed as mean±standard error of the mean, *N*=3. Different letters in superscript in the same column indicate statistically significant differences (p<0.05) according to one-way ANOVA followed by Tukey's *post hoc* test

The α-amylase inhibitory activity recorded in the present study was comparable to, and in some cases greater than, values previously reported for tea-derived polysaccharides. Pelvan *et al.* (*19*) demonstrated that black tea polysaccharides exhibited moderate α-amylase inhibition that varied depending on extraction conditions and compositional differences. Likewise, Guo *et al.* (*17*) reported significant α-glucosidase and antiglycation activities for tea polysaccharides from different tea categories, highlighting the influence of structural variability on biological performance. The comparatively lower IC_50_ values observed for Teen Ali and UPASI-28 suggest stronger inhibitory efficiency, which may be associated with their higher polysaccharide content and favourable structural characteristics.

The strong α-glucosidase inhibitory activity observed in this study supports previous findings demonstrating the antidiabetic potential of tea polysaccharides (Table 1). Guo *et al.* (*17*) reported that tea polysaccharides from pu-erh tea exhibited pronounced α-glucosidase inhibitory activity and suggested that protein-bound acidic heteropolysaccharides possess enhanced biological effectiveness. Furthermore, recent reviews have highlighted that the hypoglycaemic activity of tea polysaccharides is closely related to monosaccharide composition, molecular mass distribution and uronic acid content. The superior inhibitory activity of Teen Ali and UPASI-28 may therefore result from cultivar-dependent structural features that improve enzyme binding and interfere with carbohydrate digestion.

The IC_50_ values observed in this study (28.6−33.5 µg/mL) appear relatively low compared to those of several other polysaccharides isolated from plants, suggesting greater inhibitory efficiency. Previous investigations involving tea polysaccharides have shown that molecular structure with glycosidic linkage and uronic acid content have an important influence on biological activity. Consequently, the relatively high efficiency of Teen Ali and UPASI-28 can be attributed to their relatively low amounts of protein contamination, as confirmed by FTIR analysis. Similar investigations have also found that the biological activity of tea polysaccharides increases with structural purity (*18*).

The higher inhibitory effect of tea polysaccharides on both α-amylase and α-glucosidase could be linked to the unique physicochemical properties and structure of the polysaccharides. The presence of uronic acid units in tea polysaccharides increases the interactions between the enzymes and polysaccharides, leading to more effective enzyme inhibition. Recent evidence has shown that polysaccharides with high contents of hydroxyl and uronic acid groups exert more potent enzyme inhibition due to enhanced molecular interaction and conformational dynamics (*16*, *17*). Moreover, the macromolecular nature of polysaccharides is another factor contributing to enzyme inhibition, manifested by increased viscosity. The ability of macromolecules to form a physical barrier around starch molecules restricts the access of enzymes, resulting in slower carbohydrate hydrolysis. This process is considered critical for managing postprandial blood sugar amounts since the physical barrier delays the release and subsequent absorption of carbohydrates. Similarly, food polysaccharides with increased viscosity have been found to regulate glucose metabolism by inhibiting digestive enzymes (*19*).

### In vivo antidiabetic evaluation in alloxan-induced diabetic mice

#### Changes in body mass of diabetic mice

In the *in vivo* experimental model, the body mass of diabetic control mice decreased gradually from 28.7 g on day 0 to 22.0 g on day 21. In contrast, diabetic mice treated with tea polysaccharides showed gradual recovery in body mass. The 100 mg/kg dose of Teen Ali increased the body mass of diabetic mice to 30.5 g on day 21, while the 100 mg/kg dose of UPASI-28 produced a corresponding increase to 32.6 g on day 21, which is highly comparable to that of the metformin-treated group (33.4 g) (Table 2). These results indicate that polysaccharide supplementation is effective in preventing muscle wasting syndrome. Improved insulin sensitivity results in increased protein synthesis and, consequently, prevention of muscle wasting syndrome. Similar results were reported by El Adaouia Taleb *et al.* (*20*). One-way ANOVA with Tukey’s test showed significant differences among experimental groups (p<0.0001). The letters in superscript indicate that treatment with high doses of Teen Ali and UPASI-28 significantly increased the body mass of animals compared to the diabetic control group (p<0.05).

**Table 2:** Changes in body mass of control, diabetic and polysaccharide-treated mice during 21 days of the experiment

	*t*(treatment)/day			
Sample	0	7	14	21
	*m*(body)/g			
Control	(33.2±1.5)ᵃ	(33.8±1.4)ᵃ	(34.5±1.6)ᵃ	(35.0±1.5)ᵃ
Toxic, alloxan-induced (150 mg/kg)	(28.7±2.6)ᵇ	(26.0±2.4)ᶜ	(25.5±2.3)ᶜ	(22.0±2.2)ᶜ
Standard, metformin (150 mg/kg)	(32.5±1.7)ᵃ	(30.7±1.6)ᵇ	(33.0±1.5)ᵃ	(33.4±1.5)ᵃ
Teen Ali low dose (10 mg/kg)	(29.3±2.2)ᵇ	(27.8±2.1)ᶜ	(28.2±2.0)ᵇ	(28.8±2.0)ᵇ
Teen Ali medium dose (50 mg/kg)	(28.6±2.9)ᵇ	(26.5±2.7)ᶜ	(28.7±2.5)ᵇ	(29.9±2.4)ᵇ
Teen Ali high dose (100 mg/kg)	(27.5±2.8)ᵇ	(25.8±2.6)ᶜ	(28.2±2.4)ᵇ	(30.5±2.3)ᵇ
UPASI-28 low dose (10 mg/kg)	(29.0±2.4)ᵇ	(27.9±2.2)ᶜ	(29.6±2.1)ᵇ	(30.2±2.0)ᵇ
UPASI-28 medium dose (50 mg/kg)	(28.4±2.6)ᵇ	(27.0±2.3)ᶜ	(30.8±2.2)ᵃ	(31.7±2.1)ᵃ
UPASI-28 high dose (100 mg/kg)	(27.8±2.7)ᵇ	(26.5±2.4)ᶜ	(31.4±2.1)ᵃ	(32.6±2.0)ᵃ

Values are expressed as mean±standard error of the mean (*N*=6). Different letters in superscript in the same column indicate statistically significant differences (p<0.05) using one-way ANOVA followed by Tukey’s *post hoc* test

#### Blood glucose concentrations of diabetic mice

Blood glucose concentrations continued to rise in the diabetic control mice, reaching (450±30) mg/dL on day 21. Polysaccharide treatment induced a statistically significant hypoglycaemic effect (p<0.05) in a dose- and time-dependent manner. Treatment with Teen Ali at 100 mg/kg body mass reduced the blood glucose to (162±8) mg/dL, while UPASI-28 at the same dose showed slightly higher activity, with a final blood glucose concentration of (152±7) mg/dL (Table 3). This was almost comparable to metformin, which decreased the blood glucose to (132±15) mg/dL. ANOVA indicated statistically significant differences among the groups (p<0.0001). The Tukey’s test showed that all treatment groups differed significantly from the diabetic control group (p<0.05). High-dose groups of both plant extracts showed significantly greater differences in their glucose reduction than the low- and medium-dose groups.

**Table 3:** Fasting blood glucose concentrations measured at 0, 7, 14 and 21 days in all experimental groups

	*t*(treatment)/day			
Sample	0	7	14	21
	(*m*(glucose)/*V*(blood))/(mg/dL)			
Control	(90±6)ᶜ	(92±7)ᶜ	(89±6)ᶜ	(91±5)ᶜ
Toxic, alloxan-induced (150 mg/kg)	(320±15)ᵃ	(365±20)ᵃ	(410±25)ᵃ	(450±30)ᵃ
Standard, metformin (150 mg/kg)	(318±12)ᵃ	(270±10)ᵇ	(200±18)ᵇ	(132±15)ᵇ
Teen Ali low dose (10 mg/kg)	(310±14)ᵃ	(282±18)ᵇ	(240±16)ᵇ	(220±19)ᵇ
Teen Ali medium dose (50 mg/kg)	(315±16)ᵃ	(250±15)ᵇ	(220±12)ᵇ	(190±10)ᵇ
Teen Ali high dose (100 mg/kg)	(312±13)ᵃ	(246±12)ᵇ	(208±10)ᵇ	(162±8)ᵇ
UPASI-28 low dose (10 mg/kg)	(311±15)ᵃ	(285±17)ᵇ	(245±14)ᵇ	(209±16)ᵇ
UPASI-28 medium dose (50 mg/kg)	(314±14)ᵃ	(248±13)ᵇ	(211±11)ᵇ	(192±10)ᵇ
UPASI-28 high dose (100 mg/kg)	(313±13)ᵃ	(242±12)ᵇ	(198±9)ᵇ	(152±7)ᵇ

Values are expressed as mean±standard error of the mean (*N*=6). Different letters in superscript in the same column indicate statistically significant differences (p<0.05)

The hypoglycaemic effects observed in diabetic mice are consistent with earlier reports describing the glucose-lowering activity of tea polysaccharides. Xu *et al.* (*6*) demonstrated that oral administration of pu-erh tea polysaccharides significantly reduced blood glucose concentrations and enhanced antioxidant defence mechanisms in alloxan-induced diabetic mice. Similarly, Ren *et al.* (*10*) reported that selenium-containing tea polysaccharides improved insulin resistance and reduced oxidative stress in experimental diabetic models. Although differences in cultivar and polysaccharide composition may influence efficacy, the present findings support the growing evidence that tea polysaccharides contribute to glycaemic regulation through multiple complementary mechanisms.

#### Histopathological analysis of diabetic mice

Histopathological observations further confirmed the biochemical findings. Tissue sections from diabetic control mouse showed extensive damage to the pancreatic tissues, evidenced by the shrinking of the islets of Langerhans and disorganised structures. In contrast, tissue sections from mice treated with the polysaccharide extract exhibited well-organised pancreatic tissue structures, particularly in those that received medium and high doses of Teen Ali and UPASI-28 (Fig. 3). Tissue sections from diabetic control mice also showed extensive liver damage, characterised by steatosis, inflammation and disorganised structures. However, the treated mice exhibited well-organised structures, particularly in the tissue sections that received medium and high doses of polysaccharide extract from Teen Ali and UPASI-28. Kidney tissue sections from diabetic control mice were extensively damaged, characterised by hypertrophy and congestion, while the tissue sections from treated mice showed well-organised structures in the polysaccharide-treated groups (Fig. 4). The livers of diabetic control mice displayed significant histopathological changes, including steatosis, infiltration of inflammatory cells, and deranged hepatic architecture. In contrast, tea polysaccharide intervention significantly improved liver architecture with decreased lipid deposition and restored cellular organisation. This improvement was more evident at the medium and high doses of Teen Ali and UPASI-28, suggesting a protective role against diabetes-related liver injury (Fig. 5).

**Fig. 3:**
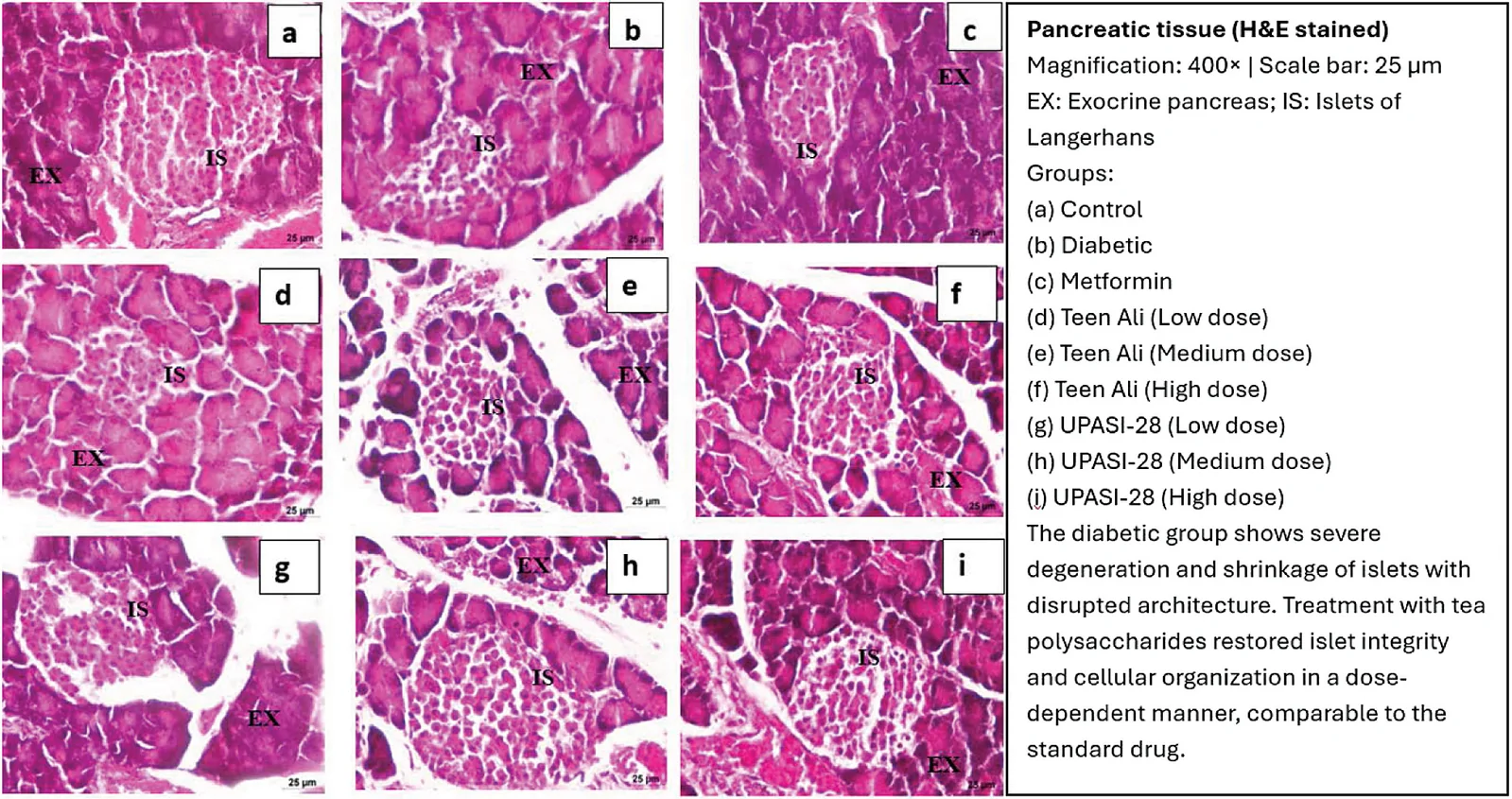
Histopathological sections of pancreatic tissue from control, diabetic and polysaccharide-treated mice showing structural alterations and restoration of islet architecture following treatment

**Fig. 4:**
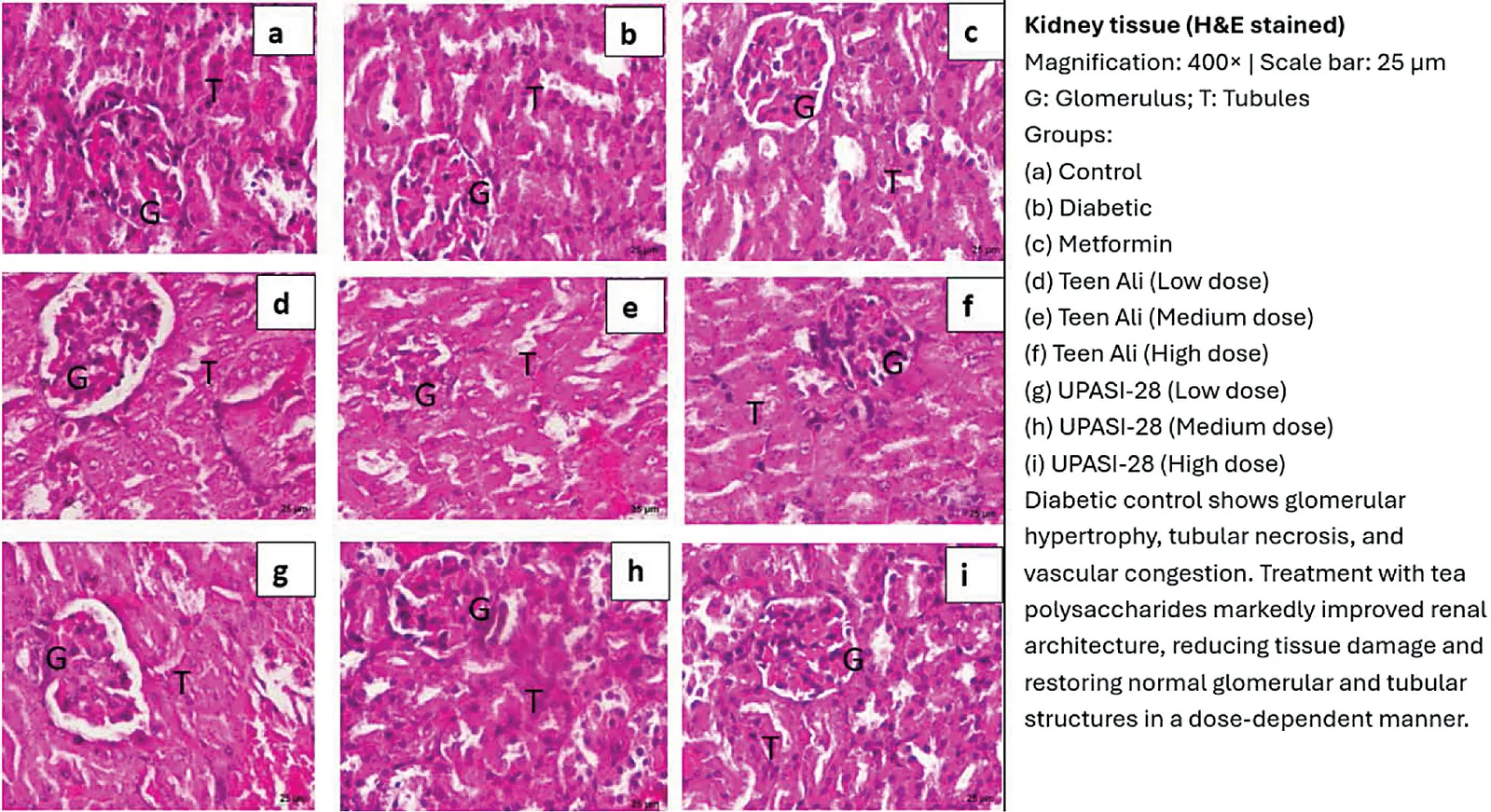
Histopathological sections of kidney tissue from control, diabetic and polysaccharide-treated mice showing tissue damage in the diabetic control and improved structural integrity after treatment

**Fig. 5:**
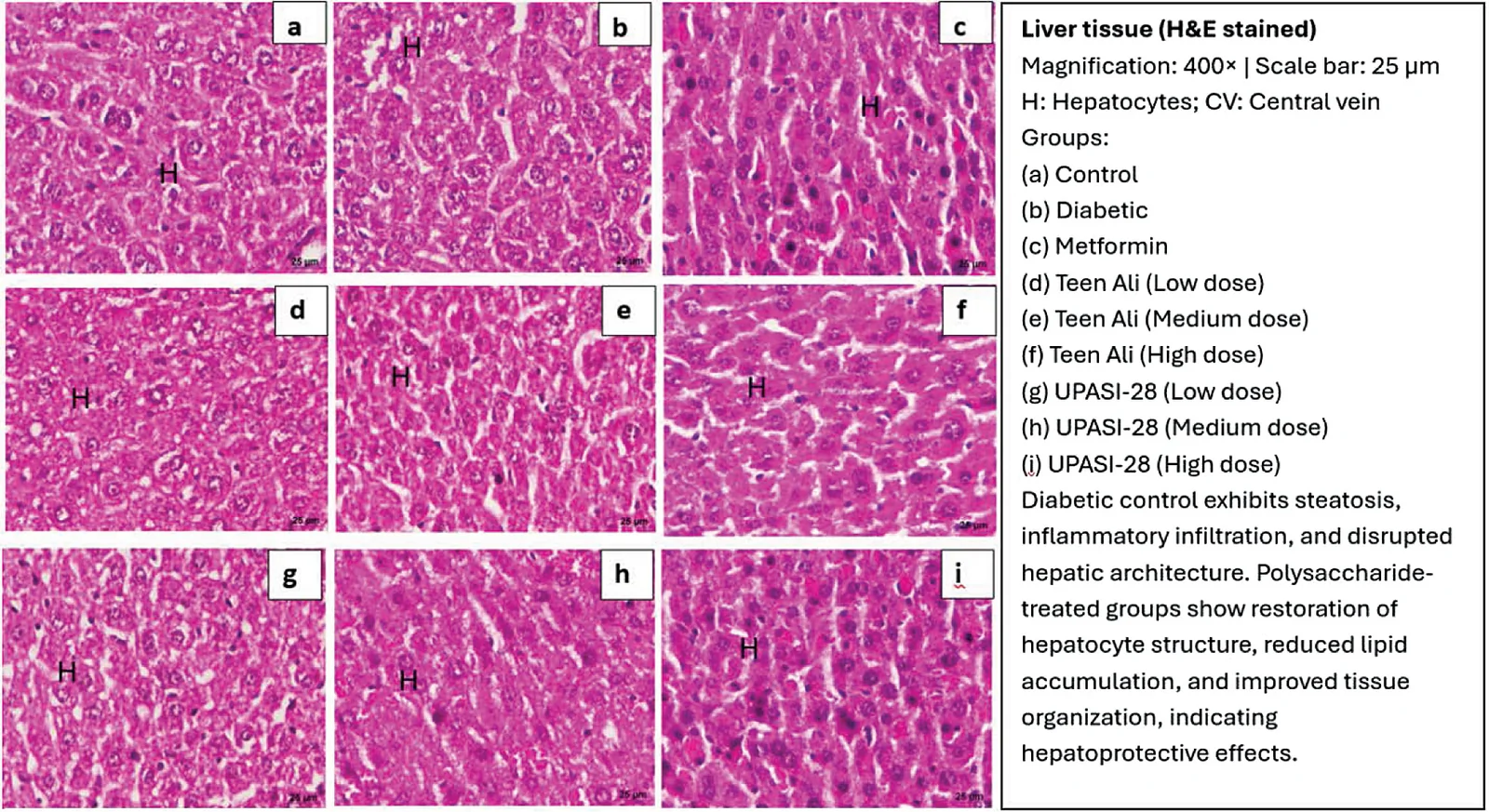
Histopathological sections of liver tissue from control, diabetic and polysaccharide-treated mice showing steatosis and inflammation in the diabetic control and restored hepatic architecture in the treated groups

Histopathological recovery of pancreatic, hepatic and renal tissues further supports the biological activity of tea polysaccharides beyond digestive enzyme inhibition. Previous studies have shown that tea polysaccharides can protect pancreatic β-cells from oxidative damage, reduce inflammatory responses, and improve tissue architecture under diabetic conditions. Wang *et al.* (*21*) reviewed evidence indicating that tea polysaccharides modulate antioxidant defence pathways and reduce reactive oxygen species accumulation, thereby limiting diabetes-associated tissue injury. The restoration of normal tissue organisation observed in the present study is consistent with these reported protective effects and suggests a broader therapeutic role for tea-derived polysaccharides in diabetes management.

The protective mechanism of tea polysaccharides may be attributed to their antioxidant activity, particularly through reactive oxygen species (ROS) scavenging and reduction of oxidative stress, which would prevent apoptosis of pancreatic β-cells and avoid subsequent damage to other organs. The antioxidant activity of natural polysaccharides has been known to involve the antiapoptotic activity of Bcl-2 and decreased levels of pro-apoptotic factors in diabetic tissues (*22*).

*In vivo* findings further support the functional efficacy of tea polysaccharides, as shown by significant reductions in fasting blood glucose concentrations, improvements in body mass, and restoration of tissue architecture in diabetic mice. These effects may be attributed to multiple mechanisms, including improved glucose utilisation, protection of pancreatic β-cells from oxidative stress, and modulation of insulin signalling pathways. Recent studies have shown that tea polysaccharides can regulate key metabolic pathways such as PI3K/Akt and improve antioxidant defence systems, thereby contributing to glycaemic control and tissue protection (*23*). The presence of statistically significant results in both *in vitro* and *in vivo* investigations (p<0.05) indicates that the biological actions of tea polysaccharides are not a result of random chance but reflect genuine pharmacological actions. The distinct statistical grouping of Teen Ali and UPASI-28 further supports cultivar-dependent variation in antidiabetic activity, likely due to differences in molecular structure and composition.

### Limitations of the study

Although this study demonstrates promising antidiabetic activity of tea polysaccharides, several limitations should be acknowledged. Structural characterisation was limited to FTIR spectroscopy and scanning electron microscopy, so important parameters such as monosaccharide composition, molecular mass distribution, glycosidic linkage patterns and quantitative uronic acid content were not determined. Consequently, the proposed structure–activity relationships should be considered preliminary. In addition, no further purification procedures were performed after extraction, and the obtained polysaccharide preparations may still contain co-extracted compounds such as proteins, polyphenols and other bioactive constituents that could contribute to the observed biological activities. Furthermore, the molecular mechanisms responsible for glucose regulation were not investigated. The biological activity was evaluated only in an alloxan-induced mouse model, and extrapolation to humans should therefore be carried out with caution. Future studies should focus on advanced structural characterisation, purification, mechanistic investigations and clinical validation.

## CONCLUSIONS

The present study demonstrated that tea polysaccharides isolated from Indian tea cultivars possess considerable antidiabetic potential. Among the six cultivars evaluated, Teen Ali and UPASI-28 yielded the highest amounts of polysaccharides and showed the strongest inhibitory activity against α-amylase and α-glucosidase. These findings were further supported by significant improvements in fasting blood glucose levels, recovery from diabetes-associated body mass loss, and restoration of tissue architecture in diabetic mice. The results highlight the importance of cultivar-dependent variation in determining the biological activity of tea polysaccharides. Nevertheless, further studies involving detailed structural characterisation, mechanistic investigations and clinical evaluation are required before their potential application as functional food ingredients or nutraceuticals can be fully established.

## SUPPLEMENTARY MATERIALS

Supplementary materials are available at: https://www.ftb.com.hr/images/pdfarticles/2026/July-September/FTB-64-324-S1.pdf.

## Figures and Tables

**Fig. S1:**
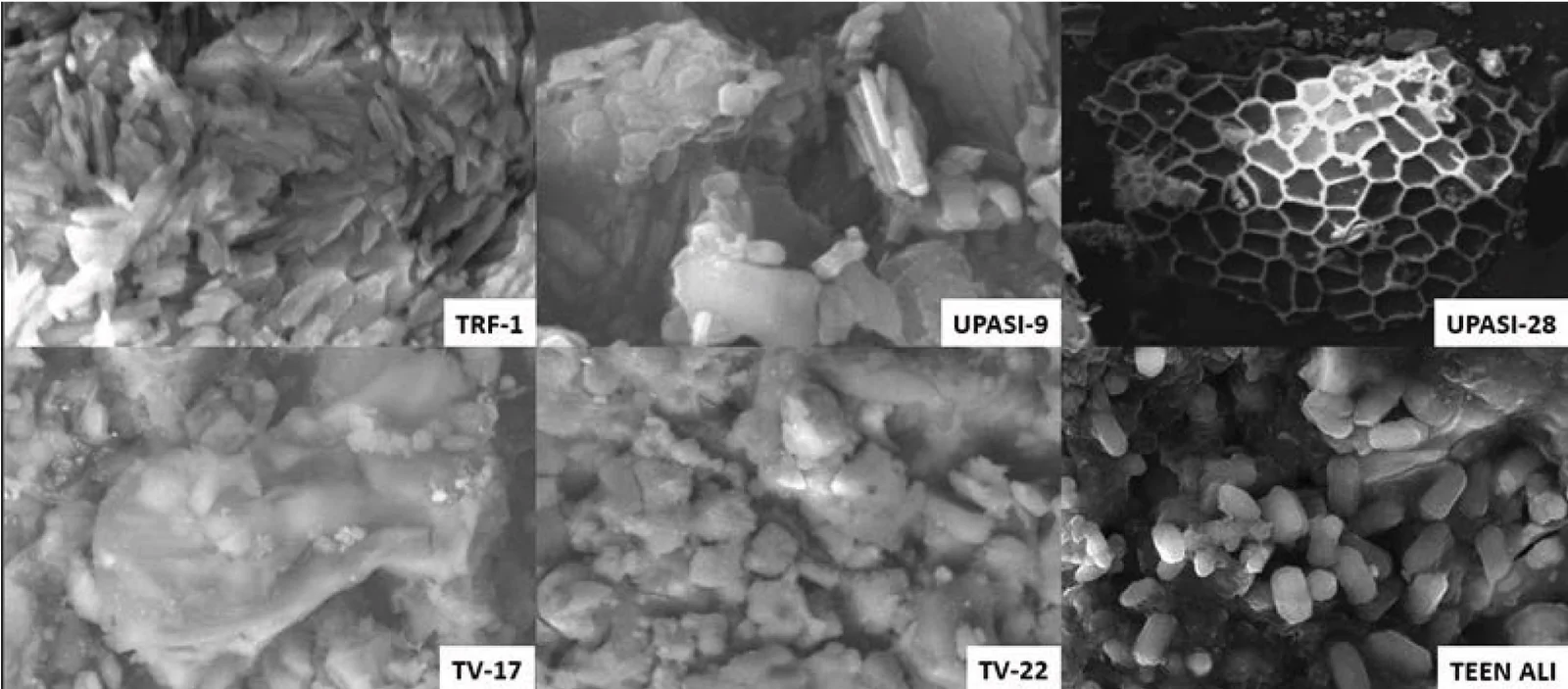
Scanning electron micrographs (250, 500, 1000 and 2500×) of tea polysaccharides from different cultivars showing variations in surface morphology, including porous, aggregated and amorphous structures
